# *ABCB1* 1199G>A Genetic Polymorphism (Rs2229109) Influences the Intracellular Accumulation of Tacrolimus in HEK293 and K562 Recombinant Cell Lines

**DOI:** 10.1371/journal.pone.0091555

**Published:** 2014-03-12

**Authors:** Géraldine Dessilly, Laure Elens, Nadtha Panin, Arnaud Capron, Anabelle Decottignies, Jean-Baptiste Demoulin, Vincent Haufroid

**Affiliations:** 1 Louvain Centre for Toxicology and Applied Pharmacology, Institut de Recherche Expérimentale et Clinique, Université Catholique de Louvain, Brussels, Belgium; 2 Louvain Drug Research Institute, Université Catholique de Louvain, Brussels, Belgium; 3 Department of Clinical Chemistry, Cliniques Universitaires Saint-Luc, Université Catholique de Louvain, Brussels, Belgium; 4 De Duve Institute, Université Catholique de Louvain, Brussels, Belgium; Indiana University School of Medicine, United States of America

## Abstract

**Objective:**

ATP-binding cassette, subfamily B, member 1 (ABCB1) transporter, or P-glycoprotein, is an efflux protein implicated in the absorption and the distribution of various compounds, including tacrolimus and cyclosporine A. *In vivo* studies suggest an association between the *ABCB1* 1199G>A single nucleotide polymorphism (SNP) and tacrolimus intracellular accumulation. The aim of the present experimental study was to clarify *in vitro* the impact of the coding *ABCB1* 1199G>A SNP on ABCB1 transport activity towards both immunosuppressive drugs.

**Method:**

Two recombinant cell lines, *i.e.* Human Embryonic Kidney (HEK293) and Human Myelogenous Leukemia (K562) cells, overexpressing ABCB1 carrying either the wild-type allele (1199G) or its mutated counterpart (1199A), were generated. The impact of the 1199G>A SNP on ABCB1 activity towards rhodamine (Rh123), doxorubicin, vinblastine, tacrolimus and cyclosporine A was assessed by accumulation, cytotoxicity and/or kinetic experiments.

**Results:**

Tacrolimus accumulation was strongly decreased in cells overexpressing the wild-type protein (1199G) compared to control cells, confirming the ability of ABCB1 to transport tacrolimus. By contrast, overexpression of the variant protein (1199A) had nearly no effect on tacrolimus intracellular accumulation whatever the model used and the concentration tested. Unlike tacrolimus, our results also indicate that cyclosporine A, Rh123 and doxorubicin are transported in a similar extent by the wild-type and variant ABCB1 proteins while the variant protein seems to be more efficient for the transport of vinblastine.

**Conclusion:**

ABCB1 encoded by the 1199G wild-type allele transports more efficiently tacrolimus in comparison to the 1199A variant protein. This observation indicates that the amino-acid substitution (Ser400Asn) encoded by the 1199A allele drastically decreases the ability of ABCB1 to drive the efflux of tacrolimus in a substrate-specific manner, in agreement with our previously published clinical data. Our study emphasizes the importance of the *ABCB1* 1199G>A polymorphism for ABCB1 activity and its potential to explain differences in drug response.

## Introduction

P-glycoprotein (P-gp) is a phosphorylated transmembrane glycoprotein of 170 kDa that mediates the ATP-dependent efflux of numerous endogenous and exogenous substances across biological membranes. P-gp is encoded in humans by the *ABCB1* gene which spans 28 exons on chromosome 7. The ABCB1 protein (official nomenclature for P-gp) belongs to the group B of the ATP binding cassette (ABC) superfamily and is composed of 1280 amino acids consisting of two equivalent and symmetric domains of 6 transmembrane segments. Each domain encloses an intracellular nucleotide binding domain (NBD), which binds ATP [1]–[4]. The expression of ABCB1 is mainly distributed in excretory organs and in the blood brain barrier [1], [5]. Consequently, ABCB1 is thought to ensure a protective role with respect to the absorption (enterocytes), the distribution (blood brain barrier, lymphocytes) and the excretion (hepatocytes, kidney) of potential harmful substances by driving their transport from the cytoplasm to the extracellular compartiment [1], [6]–[10]. Its substrates spectrum is very broad and includes important exogenous molecules, such as antiretrovirals and immunosuppressive agents (*e.g.* tacrolimus (Tac) and cyclosporine A (CsA) [11]) and also anticancer drugs (e.g. imatinib [12]). Overall, this protein influences the pharmacokinetics of various compounds and its overexpression in cancer has been suggested as a major cause of resistance to treatments [9], [10], [13]–[16].

Over the last decade more than 50 single-nucleotide polymorphisms (SNPs) have been identified in *ABCB1* [17]–[19]. The three most common SNPs in the protein coding region are rs1128503 (1236C>T, Gly412Gly), rs2032582 (2677G>T/A, Ala893Ser/Thr), and rs1045642 (3435C>T, Ile1145Ile) [20], [21]. These three SNPs are in strong linkage disequilibrium (LD) and have been extensively investigated. However, the results that have been reported so far are inconsistent and the real functional impact of these three SNPs remains controversial [22]. Other less frequent SNPs have been described and could also potentially explain part of the variability observed in the expression and/or function of ABCB1 [5]. Of particular interest, the *ABCB1* 1199G>A coding SNP located in the exon 11 (rs2229109) is relatively frequent with a reported allelic frequency of about 6% in the Caucasian population. Therefore, according to the Hardy-Weinberg distribution, the expected genotypic frequencies are respectively of 0.4% for the homozygotes mutated AA, 11.2% for the heterozygotes GA and 88.4% for the homozygotes wild-type GG. This SNP is associated with a serine to asparagine substitution at position 400 in a cytoplasmic loop of ABCB1 which is involved in substrates recognition [2]. A small number of *in vitro* studies have assessed the impact of the *ABCB1* 1199G>A SNP on the ABCB1 efflux activity towards its substrates. One study reported that cells overexpressing the variant protein (ABCB1_1199A/mut_ cells) were characterized by a decreased efflux of Rhodamine123 (Rh123), a fluorescent substrate of ABCB1, when compared to cells overexpressing the wild-type protein (ABCB1_1199G/wt_ cells). In the same study, cytotoxicity experiments using anticancer drugs have shown that ABCB1_1199G/wt_ and ABCB1_1199A/mut_ cells exhibited similar resistance to doxorubicin, while ABCB1_1199A/mut_ cells were more resistant to vinblastine and vincristine suggesting, in contrast, an increased efflux activity of the 1199A variant towards both anticancer drugs [23]. A more recent study suggested also an increased efflux activity of the 1199A variant towards several protease inhibitors (PI) used in the treatment of Human Immunodeficiency Virus (HIV) infection [24]. It can be thus rationnaly hypothesized that the impact of this SNP, may differentially affect the affinity and/or the activity of this protein in a substrate-dependent manner.

Tacrolimus (Tac) is an immunosuppressive drug, belonging to the calcineurin inhibitor group, and is widely used in kidney and liver transplantation to prevent graft rejection [25], [26]. Tac is characterized by a very narrow therapeutic window and a huge inter-individual variability in its pharmacokinetic and pharmacodynamic behavior [25], [27], [28]. Despite the use of therapeutic drug monitoring, a relatively high rate of therapeutic failure and toxicity events are still encountered. The identification of genetic markers influencing Tac pharmacokinetics and/or pharmacodynamics might potentially help the clinician to better adjust Tac therapy directly after transplantation. In this respect, we have previously linked the 1199A variant to an increased accumulation of Tac in liver biopsies [29] and in peripheral blood mononuclear cells (PBMCs) which include T-lymphocytes, the cellular target of Tac [30] and suggesting a decrease in ABCB1 mediated efflux activity towards Tac *in vivo*.

Cyclosporin A (CsA) is another powerful immunosuppressive drug, used in transplantation to prevent graft rejection but to a lesser extent than Tac partly because the concentrations used in clinic are more nephrotoxic. It was demonstrated *in vivo* that the 1199G>A SNP influences the CsA concentration in PBMCs of patients with renal transplantation [31]. Carriers of the 1199A variant presented lower intracellular levels compared to homozygotes wild-type patients, arguing for an opposite functional effect of the *ABCB1* 1199G>A SNP considering CsA-mediated efflux when compared to Tac transport.

The aim of the present experimental study was to clarify and further confirm *in vitro* the impact of the *ABCB1* 1199G>A polymorphism on ABCB1 expression and transport activity towards both immunosuppressive drugs (Tac and CsA) using two stable transfected cell lines.

## Materials and Methods

### Materials

Tac (Prograf IV) and CsA (Sandimmun) were purchased from Astellas Pharma (Brussels, Belgium) and Novartis Pharma (Vilvoorde, Belgium), respectively. LY335979 (Zosuquidar 3HCl) was purchased from Bio-connect (Huissen, Netherlands). G418 was purchased from Roche Applied Science (Vilvoorde, Belgium). Rhodamine (Rh123) was purchased from Sigma-Aldrich (St-Louis, United States). Doxorubicin and vinblastine were purchased from Pfizer (Brussels, Belgium) and Teva (Wilrijk, Belgium), respectively.

HEK293 is a cell line derived from human embryonic kidney cells grown in tissue culture (ATCC CRL-1573™). K562 is a cell line derived from the pleural effusion of a 53-year old female with chronic myelogenous leukemia in terminal blast crises (ATCC CCL-243™).

### Cell culture

HEK293 were grown in Dulbecco’s Modified Eagle medium (DMEM) 1g/l glucose and glutamine (Gibco, Invitrogen) supplemented with 10% (v/v) of Fetal Bovin Serum (FBS) (Gibco, Invitrogen) and 1% (v/v) of Antibiotic-Antimycotic solution (A-A) (Gibco, Invitrogen) at a temperature of 37°C in the presence of 5% of CO_2_. For immunofluorescence staining and intracellular accumulation assays, HEK293 cells were grown in DMEM Glutamax 4.5 g/l glucose (Gibco, Invitrogen) supplemented with 10% (v/v) FBS and 1% (v/v) A-A.

K562 were grown in Iscove’s Modified Eagle Dulbecco’s medium (IMDM) (Gibco, Invitrogen) supplemented with 10% (v/v) of FBS (Gibco, Invitrogen) and 1% (v/v) A-A (Gibco, Invitrogen) at a temperature of 37°C in the presence of 5% of CO_2_. For intracellular accumulation assay, K562 cells were grown in IMDM Glutamax (Gibco, Invitrogen), 10% (v/v) FBS and 1% (v/v) A-A.

### Generation of *ABCB1*_1199G/wt_ and *ABCB1*_1199A/mut_ plasmids

The expression vector pcDNA 3.1 with cDNA encoding *ABCB1* (*ABCB1*_1199G/wt_) was kindly provided by Dr Rodney Ho (University of Washington). The mutated plasmid designated *ABCB1*_1199A/mut_ was generated by site-directed mutagenesis using the QuickChange II XL Site-directed mutagenesis Kit (Stratagene) with the mismatched primers 5′-CAG AAA TGT TCA CTT CAA TTA CCC ATC TCG-3′ (forward) and 5′-CTT TTC GAG ATG CGT AAT TGA AGT GAA CAT-3′ (reverse). The plasmids were sequenced to confirm the presence of the mutation.

### Generation of stable recombinant cell lines

HEK293 cells were plated the day before transfection in 6 well plates (2 10^5^ cells/well) and transfected with lipofectamine 2000 (Invitrogen) according to the manufacturer’s protocol with 4 μg of plasmid DNA. To select stable transfectants, cells were replated at a low density 24h after the transfection and G418 was added 24h later at a final concentration of 0.8 mg/ml.

K562 cells (10^7^ cells) were transfected by electroporation (ECM630 device from BTX, 200 V, 75 Ω, 1300 μF) with 50 μg of plasmid DNA. To select stable transfectants, cells were replated at a low density 24h after the transfection and G418 was added 48h later at a final concentration of 1 mg/ml.

After one week on culture, cell surface ABCB1 protein expression in transfected cells (HEK293 and K562) was analyzed by flow cytometry [FITC Mouse Anti-Pglycoprotein antibody diluted 1∶10, (clone 555742, BD Pharmingen) or isotypic control diluted 1∶10 [(FITC Mouse IgG 2bk, clone 557002, BD Pharmingen) (see below)].

### Characterization of ABCB1 expression

#### Flow cytometry

10^6^ cells (HEK293 and K562) were recovered by centrifugation. Cells were washed twice with 2 ml of ice-cold HAFA solution [filtrated (0.22 μm) Hank’s buffer with 3% decomplemented FBS and NaN_3_ (20 mmol/l)]. Cells were resuspended in HAFA solution containing the primary FITC Mouse Anti-P-glycoprotein antibody diluted 1∶10 (clone17F9 557002, BD Pharmingen) or its matched isotypic control diluted 1∶10 (FITC Mouse IgG 2bk, clone27-35 555742, BD Pharmingen) and incubated 45 min on ice in the dark. Cells were further washed with 2 ml of HAFA solution, centrifugated and finally fixed in 1∶1 HAFA/paraformaldehyde (2%) (v/v). Samples were analyzed on a Fluorescence-activated cell sorting (FACS) Calibur (BD Sciences).

HEK_1199G/wt_ or K562_1199G/wt_ and HEK_1199A/mut_ or K562_1199A/mut_ cells were sorted with fluorescence parameters gated on the same level of intensity to ensure similar ABCB1 expression. The protocol was identical (see above) except the using of HAFA solution without NaN_3_.

#### Quantitative Western blot

HEK293 cells were rinsed twice with cold D-PBS (Invitrogen) and recovered with triton lysis buffer (TLB; TritonX100 0.1%, NaCl 150 mM, Tris-HCL pH 7.5 25 mM supplemented with complete protease inhibitors cocktail (Roche)) and incubated for 15 min on ice. The solution was centrifuged for 10 min, 4°C at 14000 rpm and the supernatant was recovered. The cell lysate was then sonicated for 5 min and further diluted in Laemmli sample buffer (Bio Rad, Hemel Hempstead, UK) containing 10% of β-mercaptoethanol. The cell lysate was passed five to six times through 26G needle, and samples were boiled at 60°C during 10 min to denature proteins. In order to facilitate the migration, samples were finally centrifuged 5 min, 14000 rpm at room temperature to remove cellular debris. 30 μl of each samples (40 μg of total proteins) and 15 μl of protein ladder (Multicolor High Range Protein Ladder, Fermentas) were loaded on a 7.5% Mini-Protean TGX precasted gel as recommended by the manufacturer (Bio Rad). Proteins were then transferred onto a PVDF membrane using the Novex Semi-dry blotter (VWR) at 80 mA/membrane for 120 min. Blots were blocked with bloking buffer (927-40000,LI-COR Biosciences) for at least 60 min. After several washing steps with TBS-T, membranes were incubated overnight at 4°C with a monoclonal primary antibody anti-P-gp (clone P7965, mouse, 1∶200 dilution, Sigma-Alrich) and with a monoclonal anti-calnexin antibody (ADI-SPA-860, 1∶15 000 dilution, Enzo Life Sciences) diluted in TBS-T. After three steps of washing in TBS-T, donkey anti-mouse IgG (926-32212, LI-COR Biosciences) and donkey anti-rabbit IgG (926-3223 LI-COR Biosciences) IRdye antibodies were applied to the membranes at a 1∶15000 and diluted in TBS-T for 60 min. The transferred proteins were detected using odyssey IR imaging system (LI-COR Biosciences).

#### Immunofluorescence

One day before the experiment, HEK293 cells were plated at a density of 10^5^ cells/well in complete medium. The next day, cells were rinsed with PBS/0.1% BSA, fixed with 4% of paraformaldehyde during 15 min and rinsed with PBS/0.1% BSA. Cell membranes were subsequently permeabilized with 0.1% Triton X100 during 5 min. After a washing step with PBS/0.1% BSA, cells were incubated with the primary monoclonal antibody Ab4E3 (ab10333, Abcam, 5 μg/ml, diluted with PBS/0.1% BSA) or with its isotopic control (Mouse Ig2a kappa Monoclonal, ab10353, Abcam, 25 μg/ml) for 90 min in the dark. After two washing steps with PBS and PBS/0.1% BSA, respectively, cells were incubated for 60 min with goat anti-mouse IgG coupled to FITC (ab6785, Abcam, 1 μg/ml) and with DAPI (Hoechts 33258 pentahydrate (bis-bezimide), H3569, Invitrogen). Finally, the cells were fixed once again with 4% of paraformaldehyde during 5 min. Fluorescence was analyzed in fluorescent mounting medium (Dako) with a digital microscope Evos fluorescence (AMG).

### Rh123 functional test

One day before the experiment, 10^5^ cells (HEK293 or K562) were plated in poly-L-lysine-coated black 96-well plates in complete medium. ABCB1 related transport activity was investigated by loading the cells for 60 min at 37°C with 5 μM of Rh123 in the dark. When indicated, ABCB1 inhibition was performed by pre-incubating the cells for 15 min in the presence of LY335979 (0.1 and 0.2 μM). After incubation with Rh123, the supernatants were discarded. The cells were washed two times with Dulbecco’s-PBS at 4°C and cell lysis was performed with Triton X100 1% in DMEM/or IMDM Glutamax at room temperature for 30 min for HEK293 or K562, respectively. Finally, the intracellular fluorescence of Rh123 was analyzed by a fluorimeter Spectramax Gemini Xs (Molecular Devices); excitation wavelength was set at 485 nm and emission at 530 nm.

### Thymidine incorporation assay

K562 cells were plated at 10^4^ cells/well in 96-well plates in complete medium. Cells were incubated for 24h at 37°C with varying concentrations of doxorubicin (from 10 to 2430 nM) or vinblastine (from 1.11 to 270 nM). One 1 μCi of [^3^H] thymidine was then added to each well and further incubated for 24h. The radioactivity was measured with a TopCount NXT liquid scintillation counter (PerkinElmer).

Graphics are represented by the relative proliferation (%); CPM samples/CPM control (without drugs exposure) *100.

### Tac and CsA accumulation

Tac or CsA accumulation experiments were performed according to a previously described method [32], [33], with minor modifications. One day before the experiment, 3.5 10^5^ cells (HEK293 or K562) were seeded in 24-wells plates in 500 μl of complete medium. Tac or CsA were added at six different concentrations (from 0.0015 to 0.5 μM and 0.015 to 5 μM, respectively) and cells were incubated for 120 min at 37°C, 5% of CO_2_. After incubation with Tac or CsA, the supernatant was discarded by splashing the plate and the cells were washed two times in cold PBS and detached with ice-cold PBS supplemented with 0.2% EDTA. After centrifugation at 4°C, the supernatant was discarded and cells pellets were conserved at –80°C until LC-MS/MS analysis.

### Tac intracellular kinetics

One day before the experiment, 3.5 10^5^ cells (HEK293 or K562) were seeded in 24-wells plates in 500 μl of complete medium. Tac was added at a final concentration of 0.05 μM and cells were incubated for 120 min at 37°C, 5% of CO_2_. After incubation with Tac, the supernatant was discarded by splashing the plate and the cells were allowed to efflux in serum-free medium for 15 sec, 30 sec, 1 min, 5 min, 10 min, 30 min and 1 h. At each time point (including the point measured in preliminary experiments, corresponding to 120 min of accumulation), cells were washed two times in cold PBS and detached with ice-cold PBS supplemented with 0.2% EDTA. After centrifugation at 4°C, the supernatant was discarded and cells pellets were conserved at –80°C until LC-MS/MS analysis.

### Tac quantification

Tac was quantified in cell pellets according to a previously published LC-MS/MS method [34], with minor changes. The chromatography system used was an Alliance2795 (Waters) coupled with a tandem mass spectrometer Quattro Micro (Micromass). The analytic column was a Xbridge Phenyl 3.5 μm×50 mm (Waters). Briefly, after alkalinization of cells with ammonium hydroxide 5% (v/v), Tac was extracted with chlorobutane containing the internal standard, consisting of ascomycin at a concentration of 100 ng/ml. Blank cell pellets were processed in parallel for the calibration curve (from 1.25 nM to 1.25 μM). Then, the organic phase was decanted, and evaporated to dryness. The dry residue was then reconstituted with acetonitrile and transferred to a high-performance liquid chromatography vial and 10 μl was injected into the LC-MS/MS. The absolute amount of drug present in cell extracts was normalized to the amount of protein present as assessed using the BCA kit (Thermoscientific).

### CsA quantification

We used the chromatography system and the analytic column described above for Tac quantification. CsA was extracted by precipitation with ZnSO_4_ 0.1 M in aqueous solution and with a solution of acetonitrile containing the internal standard (ascomycin 100 ng/ml). Blank cell pellets were processed in parallel for the calibration curve (from 1.25 nM to 1 μM). Then, the supernatant was recovered and transferred to a high-performance liquid chromatography vial and 10 μl was injected into the LC-MS/MS. The absolute amount of drug present in extracted cells was normalized to the amount of protein present as assessed using the BCA kit (Thermoscientific).

### Statistical analysis

All experiments were repeated minimum twice. Statistical analyses were performed by using GraphPad InStat (Version 3.05). Analyses of variance were performed under the null hypothesis that the means of the compared groups were equal. Student-Newman-Keuls tests were carried out when the differences among means were significant. P values less than 0.05 were considered as statistically significant.

## Results

### Generation of stable *ABCB1*_1199G/wt_ or *ABCB1*_1199A/mut_ transfectants

**Figure 1:**
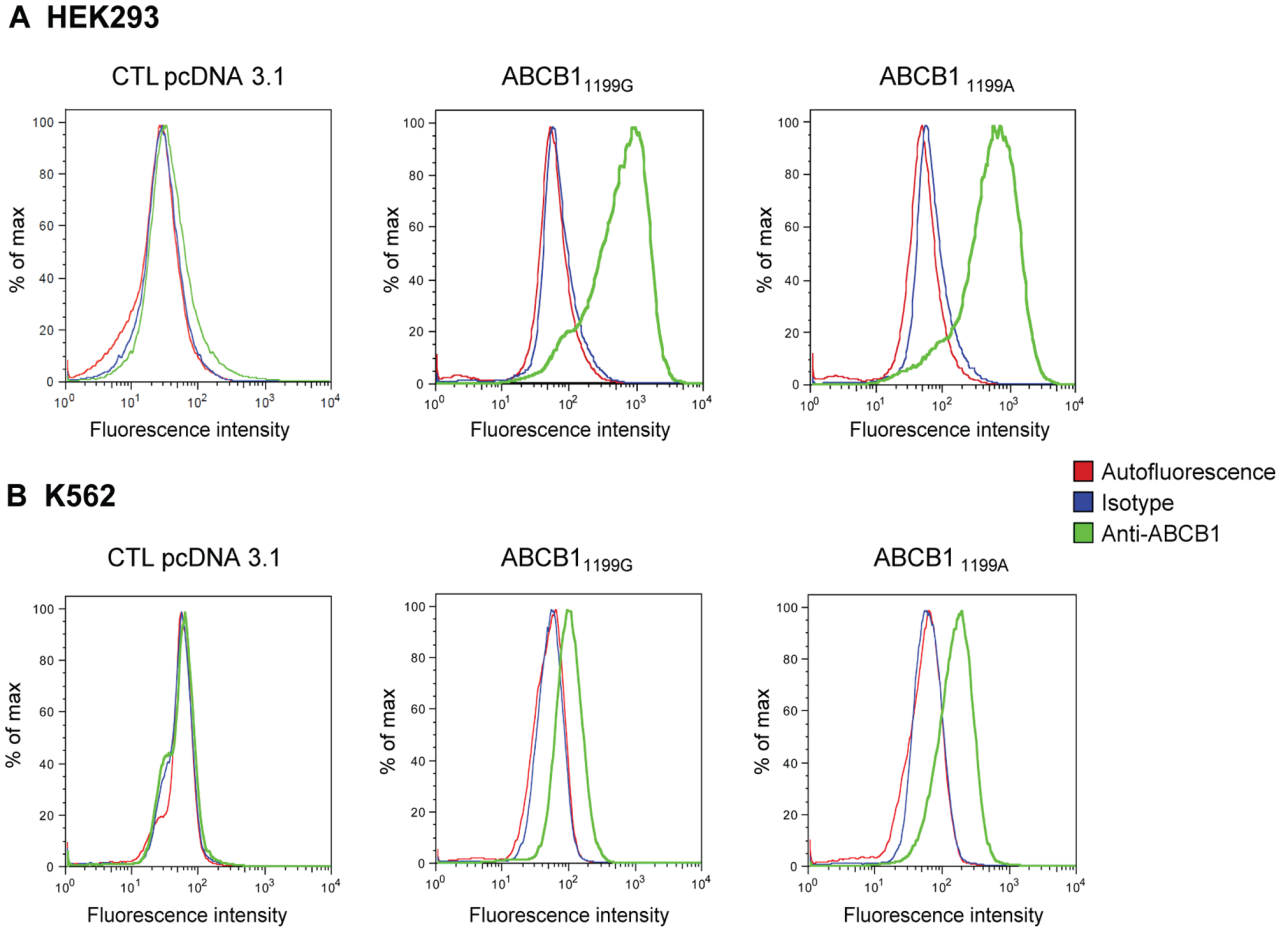
ABCB1 cell surface expression. Histograms generated from a flow cytometry analysis of (**A**) HEK293 transfected with the empty pcDNA3.1 vector (CTL HEK293 pcDNA3) and HEK293 cells transfected with *ABCB1*_1199G_ or_ 1199A_ and (**B**) K562 cells transfected with the empty pcDNA3.1 vector (CTL K562 pcDNA3.1), with *ABCB1*_1199G_ or_ 1199A_. Cells were incubated with an FITC-conjugated anti-ABCB1 antibody (green line), a matched isotypic control (blue line) or not labeled (red line).

After transfection of HEK293 or K562 cells with pcDNA3.1 empty vector, *ABCB1*_1199G/wt_ or *ABCB1*_1199A/mut_, the recombinant cell lines overexpressing ABCB1 (thereafter called HEK_1199G/wt_ or K562_1199G/wt_ and HEK_1199A/mut_ or K562_1199A/mut_, respectively) or transfected with the empty vector (thereafter called HEKpcDNA3.1 and K562pcDNA3.1) were selected in the presence of G418. Similar ABCB1 surface expression was ensured by sorting HEK_1199G/wt_ or K562_1199G/wt_ and HEK_1199A/mut_ or K562_1199A/mut_ by fluorescence activated cell sorting (FACS) with fluorescence parameters gated on the same level of intensity. As depicted in Fig 1A, comparable surface protein expression levels were confirmed by flow cytometry in both HEK_1199G/wt_ and HEK_1199A/mut_ (median fluorescence intensity 644 versus 583 arbitary units (AI), respectively). No fluorescence signal was detected in the transfected with the HEKpcDNA3.1 cell line, suggesting a very weak endogenous expression. Even though cells were similarly sorted based on the fluorescence signal emitted after ABCB1 surface labeling, K562_1199G/wt_ still show a slightly lower protein expression signal when compared to K562_1199A_ (Fig 1B) (median fluorescence intensity 99 versus 161 AI, respectively) as assessed by our second flow cytometry analysis on sorted cells. It is worth noticing that our results indicated a substantially lower level of ABCB1 surface expression in K562 than in HEK293 recombinant cell lines (Fig 1A and B).

**Figure 2:**
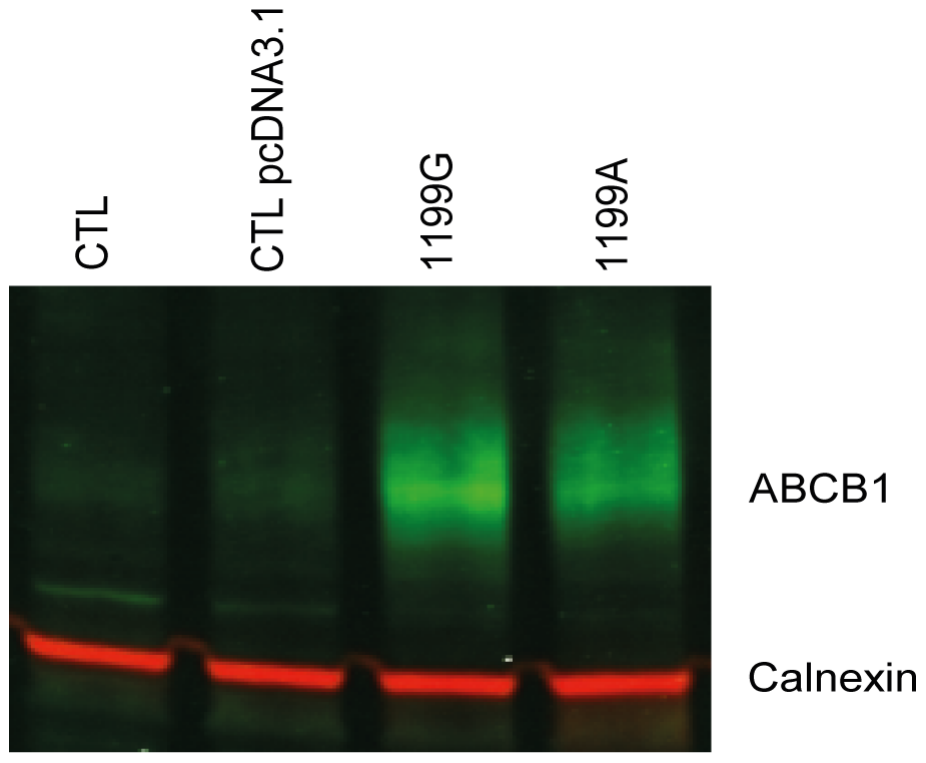
ABCB1 expression analysis by western blot. (**A**) Untransfected (CTL) HEK293, (**B**) transfected with empty plasmid (CTL pcDNA3.1) HEK pcDNA3.1 (**C**) HEK_1199G_ and (**D**) HEK_1199A_ cells lyzed. Proteins extracts were analyzed by western blot using two color odyssey system with anti-ABCB1 (green) and anti-calnexin (red) antibodies as described in materials and methods.

The expression level of ABCB1 was also examined by western blot analysis of total cell extracts of HEK293 cell lines (Fig 2). The results confirmed that the recombinant cell lines overexpressed ABCB1 efflux protein and further suggested that the total cellular expression of ABCB1 was somethat higher in HEK293 expressing the wild-type protein when compared to its variant counterpart (intensity ratio 68% compared to wild-type protein) (Fig 2).

**Figure 3:**
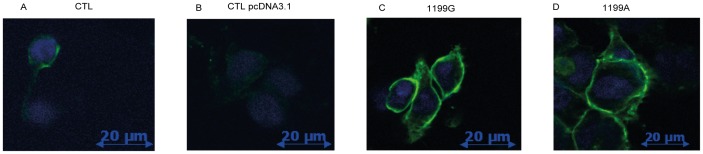
ABCB1 expression analysis by fluorescence microscopy. (**A**) Untransfected (CTL) HEK293, (**B**) transfected with empty plasmid (CTL pcDNA3.1) HEK pcDNA3.1 (**C**) HEK_1199G_ and (**D**) HEK_1199A_ cells were stained with anti-ABCB1 antibodies (green fluorescence) as described in materials and methods. DAPI was used to stain nuclei (blue).

The cellular localization of wild-type and variant ABCB1 proteins was evaluated by immunofluorescence staining of HEK293 cell lines (Fig 3). A strong circular fluorescent staining was observed in both HEK_1199G/wt_ and HEK_1199A/mut_ cells (Fig 3C and 3D) and indicated a dominant membrane localization of ABCB1.

### Impact of *ABCB1* 1199G>A polymorphism on the intracellular accumulation of Rh123

**Figure 4:**
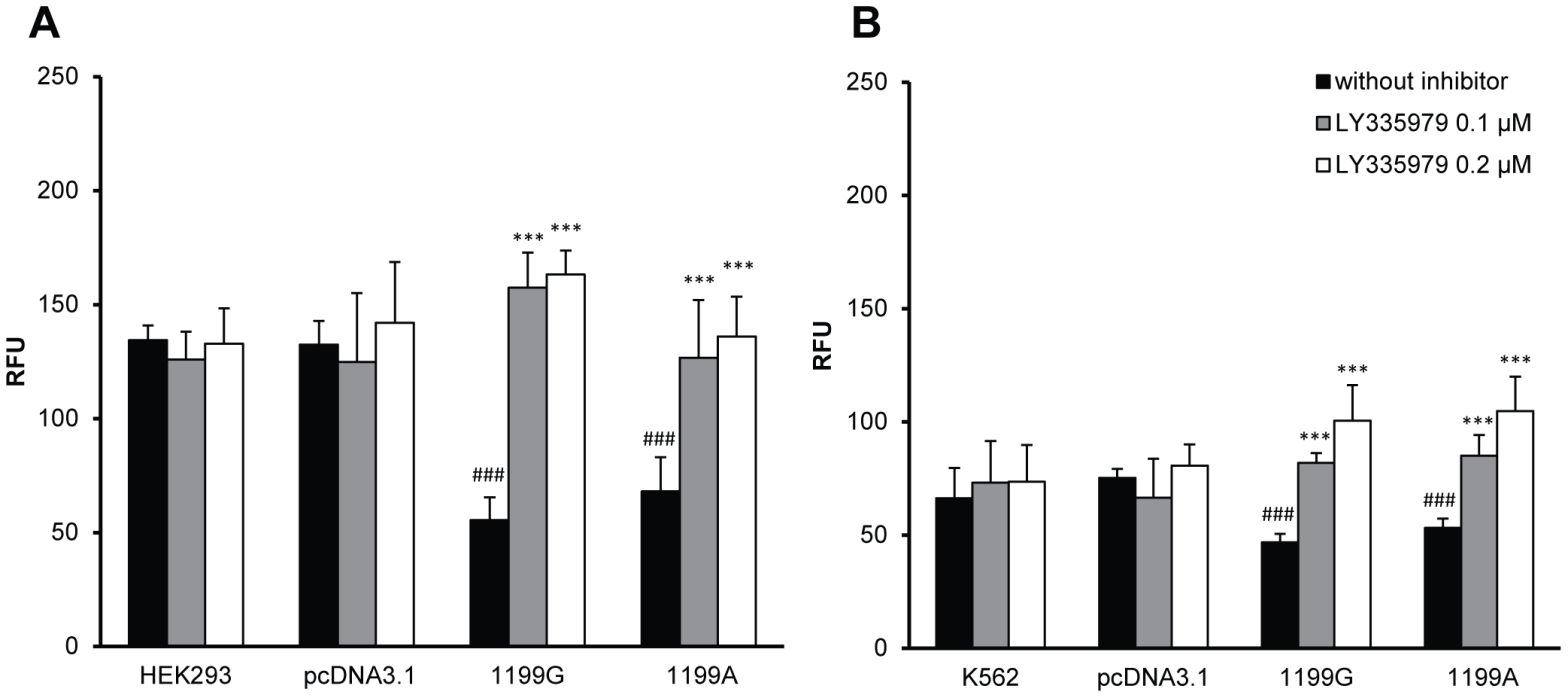
Intracellular accumulation of Rhodamine123 is not influenced by *ABCB1* 1199A variant. Intracellular accumulation of Rhodamine123 (5 μM) in presence (0.1 and 0.2 μM) or absence of ABCB1 inhibitor (LY335979) (N = 6) in (**A**) CTL HEK293, CTL HEKpcDNA3.1, HEK_1199G_ and HEK_1199A_ and (**B**) CTL K562, CTL K562pcDNA3.1, K562_1199G_ and K562_1199A_. * compared without inhibitor *p<0.05 **p<0.01 ***p<0.001, # compared to CTL pcDNA3.1 #p<0.05 ##p<0.01 ###p<0.001.

We further assessed the ability of the 1199A variant to export Rh123, a well characterized fluorescent substrate of ABCB1. As expected, after 90 min of incubation in the presence of 5 μM Rh123, fluorescence levels were lower in both HEK_1199G/wt_ and HEK_1199A/mut_ cell lines compared to control cell lines (Fig 4A, p<0.001), indicating higher Rh123 efflux in ABCB1 recombinant cells. As a control, inhibition by LY335979 (0.1 and 0.2 μM), which is a potent specific inhibitor of ABCB1-mediated efflux, yielded uniform Rh123 intracellular fluorescence values in the two transfected cell lines (Fig 4A, p<0.001), suggesting that the observed differences in fluorescence intensity can be ascribed to a higher ABCB1 efflux activity in HEK_1199G/wt_ and HEK_1199A/mut_. These experiments also confirmed that the ABCB1 recombinant protein expressed in HEK293 cells was functionally active, at least towards Rh123. Similarly, when performing Rh123 accumulation assay in K562 cells, we observed a lower fluorescence level in both K562_1199G/wt_ and K562_1199A/mut_ cell lines compared to control cell lines (Fig 4B, p<0.001). These differences were abolished when ABCB1-mediated efflux was inhibited by LY335979 (Fig 4B, p<0.001), again suggesting that recombinant proteins were functionally active and correctly inhibited by LY335979.

### Impact of *ABCB1* 1199G>A polymorphism on the cytostatic effects of doxorubicin and vinblastine

**Figure 5:**
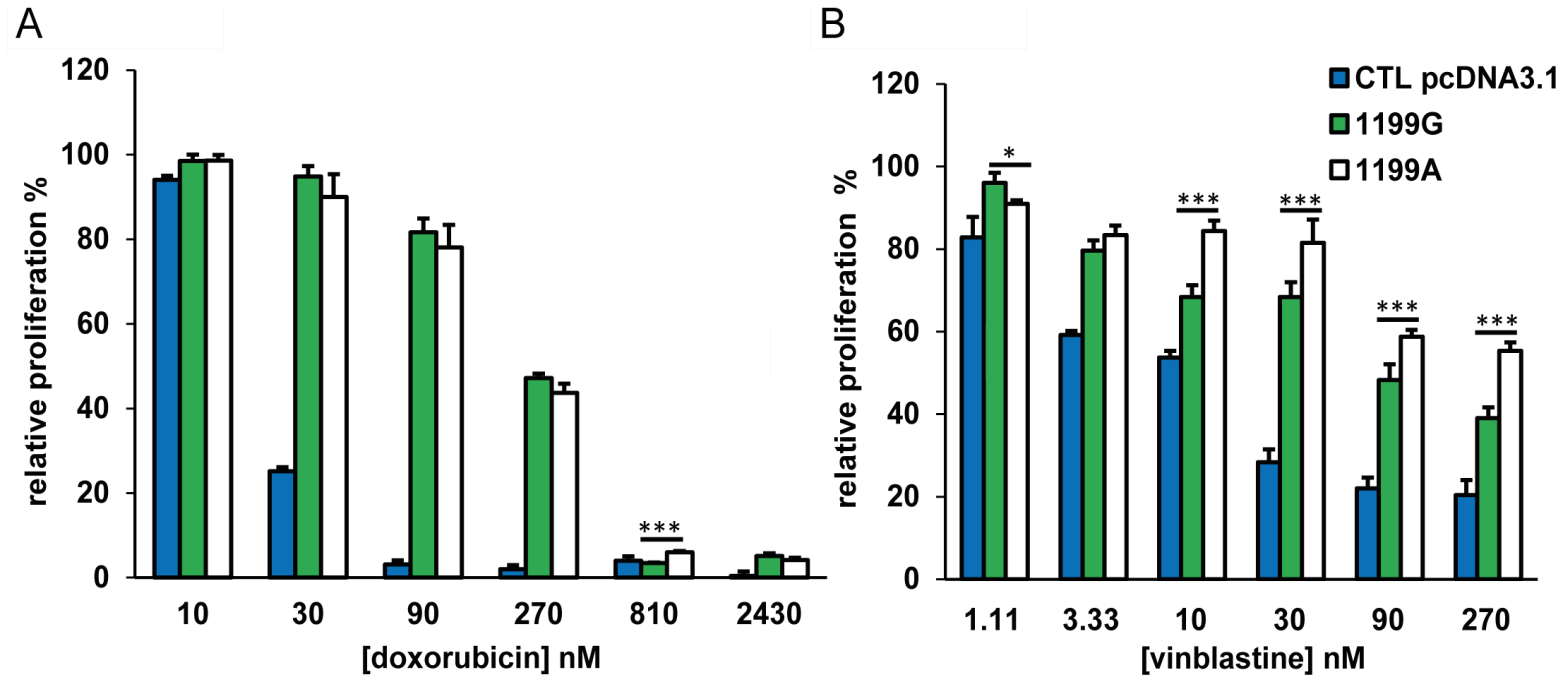
The *ABCB1* 1199A variant differentially affects cytotoxicity to doxorubicin and vinblastine. Cell proliferation after 24h of exposure (N = 4) at six different concentrations of (A) doxorubicin (10–2430 nM) or (B) vinblastine (1.11–270 nM). Graphics are represented by the relative proliferation (%); CPM samples/CPM control (without drugs exposure) *100. * compared to 1199G/WT *p<0.05 **p<0.01 ***p<0.001.

Since ABCB1 has been reported to transport doxorubicin and vinblastine [35]–[37], we assessed the influence of ABCB1 overexpression and of the variant 1199A on K562 cells proliferation in the presence of these cytostatic drugs, to further validate our models. We observed that recombinant models (K562_1199G/wt_ and K562_1199A/mut_) are resistant to both anticancer drugs, doxorubicin and vinblastine (Fig 5A, p<0.05, Fig 5B, p<0.01 statistics not presented in the figure for clarity). Furthermore, K562_1199G/wt_ and K562_1199A/mut_ cells exhibited similar resistance to doxorubicin (Fig 5A, p>0.05), while K562_1199A/mut_ cells were more resistant to vinblastine (Fig 5B, p<0.05) compared to K562_1199G/mut_. This result can be interpretated as an increase of efflux activity of the 1199A variant towards vinblastine and is in agreement with previously published results [13], [23].

### Impact of *ABCB1* 1199G>A polymorphism on the intracellular accumulation of Tac and CsA

As it has been previously shown that Tac accumulation in PBMCs and in hepatocytes was correlated with the *ABCB1* 1199G>A polymorphism *in vivo* [29], [30], we sought to determine whether this polymorphism could affect the accumulation of Tac in HEK293 and K562 transfected cell lines. HEK293 transfected cell lines were loaded for 120 min with six different concentrations of Tac (from 0.0015 to 0.5 μM) that largely cover the entire range encountered in clinical practice (the whole-blood through level C_min_ being 0.005 μM and the peak concentration C_max_ 0.016 μM). K562 cells were only incubated in presence of 0.05 μM of Tac, because this concentration roughly corresponds to the double C_max_ value and is thus assumed to be of clinical relevance.

**Figure 6:**
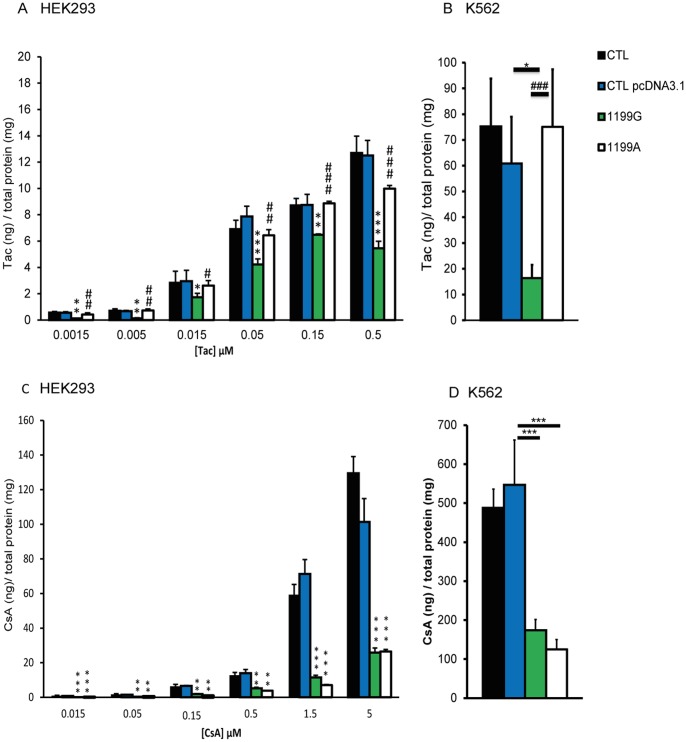
The *ABCB1* 1199A variant differentially affects Tac and CsA efflux. Intracellular accumulation of tacrolimus after 120 min of incubation (N = 3) (**A**) at six different concentrations (0.0015–0.5 μM) in CTL HEK293, CTL HEKpcDNA3.1, HEK_1199G_ and HEK_1199A_ and (**B**) at 0.05 μM in CTL K562, CTL K562 pcDNA3.1, K562_1199G_ and K562_1199A_. Intracellular accumulation of CsA after 120 min of incubation (N = 3) (**C**) at six different concentrations (0.015–5 μM) in CTL HEK, CTL HEKpcDNA3.1, HEK_1199G_ and HEK_1199A_ and (**D**) at 0.5 μM in CTL K562, CTL K562pcDNA3.1, K562_1199G_ and K562_1199A_. The intracellular accumulation of Tac or CsA in each cell line was normalized by reporting the absolute amounts of Tac or CsA (in ng) on the total amount of proteins in cell extracts, expressed in mg. * compared to 1199G/WT *p<0.05 **p<0.01 ***p<0.001.

As depicted in Fig 6A and 6B, Tac intracellular concentrations were strongly decreased in cells overexpressing the 1199G wild-type allele when compared to control cells (*i.e.* transfected with empty plasmid) (Fig 6A, p<0.01; Fig 6B, p<0.05) or to cells overexpressing the 1199A variant allele (Fig 6A, p<0.01; Fig 6B, p<0.001), and this effect was observed whatever the concentration engaged in the experiment (Fig 6A) or the model used. Overexpression of the 1199A variant allele had no effect on Tac accumulation when compared to control cell lines (p>0.05), suggesting that substitution of serine 400 for asparagine strongly reduces ABCB1-mediated Tac efflux in HEK293 and K562 cell lines.

As it was also suggested that CsA concentrations in PBMCs were correlated with *ABCB1* 1199G>A polymorphism *in vivo* [31], we similarly tested the impact of the *ABCB1* 1199G>A SNP on CsA accumulation. Six different concentrations were tested (from 0.015 to 5 μM) using HEK293 cell line in order to cover the range of concentrations encountered in patients (the whole-blood through level C_min_ being 0.03 μM and the peak concentration C_max_ 0.21 μM). For K562, we selected a clinically relevant concentration of 0.5 μM (which corresponds to the double C_max_ value). After 120 min of drug accumulation at 0.5 μM, CsA intracellular concentrations were decreased in 1199G/WT cells and in 1199A/mut cells when compared to control cells in HEK293 or K562 cell lines (Fig 6C, p<0.01; Fig 6D, p<0.001) but no difference (p>0.05) was observed between the 1199G/WT and 1199A/mut cells. Similar results were observed when incubating HEK293 cells with other tested CsA concentrations (Fig. 6C).

For intracellular kinetics involving Tac, we finally loaded cells using a Tac concentration of 0.05 μM (see above). Both transfected cell lines were thus loaded with Tac (0.05 μM) for 120 min and then placed in a Tac-free medium for up to 60 min to allow for efflux.

**Figure 7:**
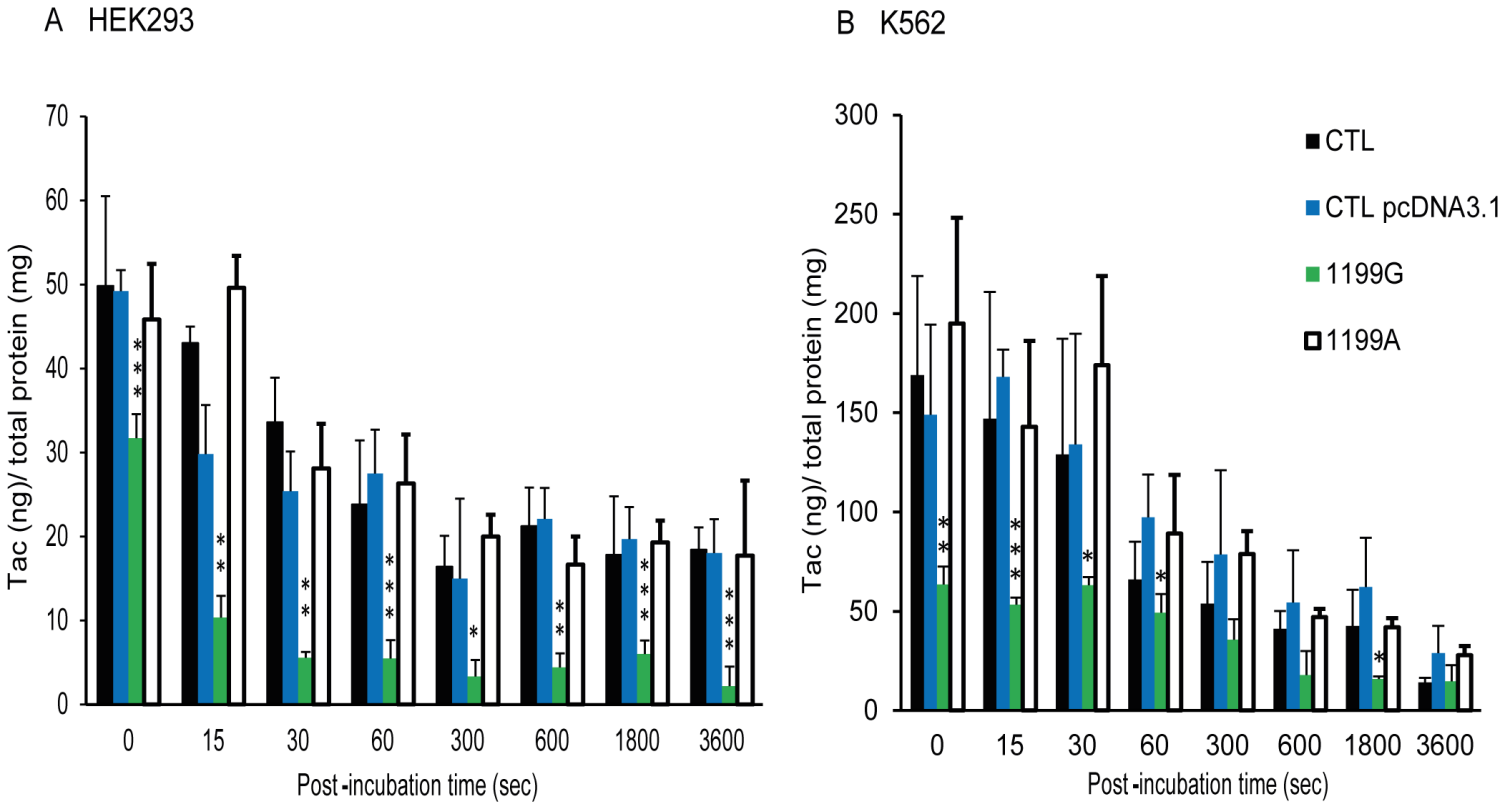
Tacrolimus intracellular kinetics. Intracellular accumulation of tacrolimus (0.05 μM) after 15, 30 sec, 1, 5, 10, 30 or 60 min of efflux (N = 6) in (**A**) CTL HEK293, CTL HEKpcDNA3.1, HEK_1199G_ and HEK_1199A_ and (**B**) CTL K562, CTL K562pcDNA3.1, K562_1199G_ and K562_1199A_. Cells were pre-loaded with tac for 120 min before efflux. The intracellular accumulation of Tac in each cell line was normalized by reporting the absolute amounts of Tac (in ng) on the total amount of proteins in cell extracts, expressed in mg. * compared to CTL pcDNA3.1 *p<0.05 **p<0.01 ***p<0.001, # compared to 1199G/WT #p<0.05 ##p<0.01 ###p<0.001.

As depicted in Fig 7, Tac intracellular concentrations were strongly decreased, at all timepoints in cells overexpressing the 1199G wild-type allele when compared to control cells (transfected with empty vector) (Fig 7A, p<0.01) or to cells overexpressing the 1199A variant allele (Fig 7A, p<0.01; Fig 7B, p<0.01 statistics not presented in the figure for clarity). Overexpression of the 1199A variant allele had no effect on Tac accumulation when compared to control cell lines (p>0.05), suggesting that the ABCB1_1199A_ variant abolished ABCB1-mediated Tac efflux in both HEK293 and K562 cell lines.

## Discussion

To assess the functional relevance of the *ABCB1* 1199G>A polymorphism, we have generated stable HEK293 and K562 cells lines overexpressing either the 1199G wild-type or the 1199A variant allele. Importantly, the basal expression of endogenous ABCB1 was negligible in these cells, which present the 1199GG genotype (genotyping results not shown). Using flow cytometry and fluorescence microscopy, we also showed that the transfected ABCB1 protein was properly expressed at the plasma membrane. Basically, our western blot results indicated that the total cellular expression of ABCB1 is slightly higher in HEK293 expressing the wild-type protein when compared to its variant counterpart. However, even if existent, the difference observed in total cell extract expression does not change the interpretation of our accumulation results (see below) as only the protein that is addressed to the membrane is supposed to be active in terms of drug efflux. In fluorescence flow cytometry assays performed on these cell lines, it appeared that the levels of ABCB1 surface expression was the same when we compared HEK293 cells expressing the wild-type with cells expressing the variant protein and this was supported by our immunofluorescence microscopy results. Consequently, our results highlight that the wild-type and the variant cell line were comparable when considering the surface protein pool implicated in the efflux of ABCB1 substrates and that our model is suitable for the analysis of the intrinsic impact of the *ABCB1* 1199G>A SNP. To test the functionality of the expressed protein in our cell lines and further validate our models, we first analyzed the rate of accumulation of Rh123. We observed that control cells had a higher Rh123 rate of accumulation compared to both transfected cell lines (*ABCB1*_1199G/wt_ and *ABCB1*_1199A/mut_) regardless of the model used, *i.e* HEK293 or K562. Altogether, our expression as well as functionality test results confirmed that ABCB1 protein is expressed similarly on the surface of recombinant cell lines and is functionally active. We also showed that Rh123 accumulation in control cells was not influenced by the presence of the LY335979 inhibitor, consistent with the very low basal expression of ABCB1 in our models. By contrast, and as expected, Rh123 accumulation in transfected cell lines was sensitive to the LY335979 inhibitor.

Moreover, we made cytotoxicity experiments using doxorubicin and vinblastine, to test whether the 1199A variant influenced the sensitivity of our recombinant models towards these two anticancer drugs. We observed that controls cells had a higher sensitivity to doxorubicin and vinblastine, thereby confirming that recombinant models transport both drugs outside of the cell more effliciently than cells not overexpressing ABCB1. We showed also that K562_1199G/wt_ and K562_1199A/mut_ cells exhibited similar resistance to doxorubicin, while K562_1199A/mut_ cells were more resistant to vinblastine compared to K562_1199G/wt_. This result suggests an increase of efflux activity of the 1199A variant towards vinblastine but no towards doxorubicin. These observations are in agreement with a previous study published by Woodahl *et al*. [13] (see below in discussion) further validating our model.

The key finding of the present study highlights that the *ABCB1* 1199G>A polymorphism has a marked influence on the accumulation of Tac in transfected cultured cell lines. First, we observed that Tac intracellular concentrations were much reduced by overexpressing ABCB1 whatever the drug concentration tested or the model used, confirming that Tac is indeed a good ABCB1 substrate [11]. Furthermore, Tac intracellular concentrations in cells overexpressing the variant ABCB1_1199A_ protein were significantly higher than in cells overexpressing wild-type ABCB1_1199G_. These results provide an important molecular clarification of our previous *in vivo* results [29], [30]. Indeed, the 1199A variant was previously linked to a decreased ABCB1 activity towards Tac *in vivo*, as reflected by the observation that carriage of the 1199A variant allele is associated with an increased Tac accumulation in PBMCs or hepatocytes isolated from renal or hepatic transplant patients, respectively [29], [30]. The present study suggests a more severe decrease of *ABCB1* 1199A activity towards Tac than observed *in vivo*. However, one should keep in mind that *in vivo,* some possible compensatory mechanisms, probably involving other ABC efflux proteins, might counterweight this loss of activity and, consequently, limit the impact of the *ABCB1* 1199A SNP considering *in vivo* Tac pharmacokinetics. In sharp contrast to Tac data, we observed a similar efflux of CsA in cells expressing the 1199A protein when compared to cells expressing the 1199G protein. Such discrepancy between Tac and CsA has been also observed *in vivo* where lower CsA concentrations were reported in PBMCs of renal transplant patients carrying the 1199A variant allele [31]. These results suggest thus an opposite effect of the *ABCB1* 1199G>A SNP on the transport of CsA and Tac.

The differential activity of *ABCB1* 1199G>A SNP towards Rh123, doxorubicin, vinblastine, Tac and CsA could be explained by the fact that the ABCB1 Ser400Asn substitution is located in a cytoplasmic loop involved in substrate recognition. This variant could thus affect differentially the affinity of ABCB1 towards its specific substrates. This hypothesis is thus corroborated when looking at the differential results observed with the substrates investigated in our study. Indeed, our results suggest a decreased activity of the variant protein towards Tac while it seem to indicate no or reverse effect for doxorubicin, CsA, Rh123 and vinblastine. These *in vitro* observations reinforce our initial hypothesis that the effect of the 1199A variant depends on the investigated substrates.

It is interesting to stress that no difference in Rh123 export was observed between *ABCB1*_1199G/wt_ and *ABCB1*_1199A/mut_ transfected cell lines. Indeed, these observations seem *a priori* in contradiction with the study by Woodahl *et al* suggesting that cells overexpressing the mutated protein (1199A) exhibited a higher accumulation rate of Rh123 compared to cells overexpressing wild-type protein (1199G) [23]. Some differences in study design could explain those discrepencies. First, a different cell model was used: the porcine kidney epithelial LLC-PK1 cell line which consists of differentiated and polarized cells. Secondly, the experimental approach was different as Woodahl *et al.* analyzed the transmembrane directional transport mediated by ABCB1 while we analyzed the intracellular accumulation. This could potentially lead to differences in sensitivity to detect changes in ABCB1 activity towards Rh123.

Interestingly, the biological impact of the *ABCB1* 1199G>A polymorphism seems not limited to tacrolimus and vinblastine. This SNP was also reported to influence the pharmacokinetic and/or pharmacodynamic parameters of antiretrovirals and other anticancer agents. Woodahl *et al.* demonstrated *in vitro* that, for all investigated antiretroviral protease inhibitors (Amprenavir, Indinavir, Lopinavir, Ritonavir and Saquinavir), the transepithelial permeability ratio was significantly greater in *ABCB1*_1199A/mut_ than in *ABCB1*_1199G/wt_ LLC-PK1 cells, reflecting a higher efflux of the drugs across the epithelial barrier. These observations suggest that the *ABCB1* 1199G>A SNP increases the ability of ABCB1 to transport antiretroviral PIs and that it might decrease their oral bioavailability and their penetration into cells and tissues [24]. However, the clinical relevance of this observation has still to be confirmed by clinical studies.

Another *in vitro* study published by Woodahl *et al.* evaluated the potential alterations in ABCB1-mediated transport of anticancer agents in relation to the *ABCB1* 1199G>A SNP [13]. The authors demonstrated that the recombinant (LLC-PK1) cells overexpressing either the variant or the wild-type protein displayed comparable doxorubicin resistance, suggesting no change in ABCB1 activity towards this drug. However, the *ABCB1* 1199A variant cells displayed greater resistance to vinblastine (as in our study), vincristine, paclitaxel, and etoposide (VP-16) in comparison to 1199G expressing cells, illustrating a potential increase in ABCB1 activity related to the variant 1199A allele towards those drugs. Taken together, these studies indicate that the effect of the *ABCB1* 1199G>A SNP seems to be substrate-specific (Table 1). Indeed, while wild-type ABCB1_1199G_ appears to be more efficient towards Tac, the *ABCB1*_1199A_ variant would be more active towards CsA, PIs (Amprenavir, Indinavir, Lopinavir, Ritonavir and Saquinavir) and four anticancer drugs (Vinblastine, Vincristine, Paclitaxel and Etoposide). As mentioned above, this hypothesis is supported by the fact that this coding SNP leads to an amino-acid substitution which is located close to the substrate binding site [5], [10]. All these interesting considerations substantiate the hypothesis of a substrate-dependent effect of the *ABCB1* 1199G>A SNP on the ABCB1 ability to transport its substrates. However, it appears clear that, for anti-cancer drugs in particular, these *in vitro* observations have to be validated from a pharmacokinetic point of view and obviously warrant further *in vivo* association studies.

**Table1:** Influence of *ABCB1* 1199G>A polymorphism on drug transport and/or efficacy.

Compound	1199A activity* (in vitro assay)	Clinical observations in 1199A carriers	References
Rhodamine	= or ↓ (fluorescence)	n.a.	23 & herein
Doxorubicin	= (cytotoxicity)	n.a.	13 & herein
Vinblastine	↑ (cytotoxicity)	n.a.	13 & herein
Vincristine	↑ (cytotoxicity)	n.a.	13
Paclitaxel	↑ (cytotoxicity)	n.a.	13
Etoposide (VP-16)	↑ (cytotoxicity)	n.a.	13
HIV protease inhibitors	↑ (accumulation)	n.a.	24
Cyclosporin A	= or ↑ (accumulation)	Lower concentration in PBMCs	31 & herein
Tacrolimus	↓ (accumulation)	Higher concentration in PBMCs and hepatocytes	& herein

*Compared to 1199G wild-type; n.a.: not available.

See text for details.

It is also important to mention the potential relevance of other *ABCB1* SNPs. Indeed, the three most common SNPs in the protein coding region are rs1128503 (1236C>T, Gly412Gly), rs2032582 (2677G>T/A, Ala893Ser/Thr), and rs1045642 (3435C>T, Ile1145Ile) [18]–[19]. The three 1236C>T, 2677G>T and 3435C>T *ABCB1* SNPs that are in high LD are particularly relevant to study from a clinical and therapeutic point of view. These SNPs are not only present at a high allelic frequency in the Caucasian population (about 50%) but have been also reported to have functional consequences on the activity of ABCB1 [20]. For instance, the *ABCB1* 3435C>T SNP has been associated with reduced ABCB1 expression and/or activity in the liver, the intestine, the kidney and the lymphocytes [9], [21]. Moreover, several *in vitro* studies using recombinant expression models were performed and seem to confirm an impact of these three SNPs on ABCB1 transport activity and suggested an influence of these SNPs on pharmacokinetics of drugs [38], [39]. However, until now, the *in vivo* impact of these three SNPs remain controversial and must be further investigated [22].

In summary, and in line with previous clinical observations, our *in vitro* results demonstrate that *ABCB1* 1199G>A SNP strongly decreases the ability of ABCB1 to transport Tac across cellular membranes, in sharp contrast to CsA. Our results also confirm the importance to investigate the true clinical significance of this SNP for Tac therapy and to assess its potential impact on the clinical outcome (*i.e*. graft rejection and toxicity) in prospective clinical trials. Finally, our study emphasizes the importance of systematically including the *ABCB1* 1199G>A SNP in every pharmacogenetic investigation involving ABCB1 substrates, especially those showing intracellular activity and/or toxicity.
