# Correction: *ABCB1* 1199G>A Genetic Polymorphism (Rs2229109) Influences the Intracellular Accumulation of Tacrolimus in HEK293 and K562 Recombinant Cell Lines

**DOI:** 10.1371/journal.pone.0112519

**Published:** 2014-10-31

**Authors:** The PLOS ONE Staff

There is an error in the first sentence of the final paragraph of the “Impact of ABCB1 1199G>A polymorphism on the intracellular accumulation of Tac and CsA” subsection. The sentence should read:

“As depicted in Fig 7, Tac intracellular concentrations were strongly decreased, at all timepoints in cells overexpressing the 1199G wild-type allele when compared to control cells (transfected with empty vector) (Fig 7A, p<0.05) or to cells overexpressing the 1199A variant allele (Fig 7A, p<0.01; Fig 7B, p<0.01 statistics not presented in the figure for clarity).”

In Table 1, the final entry under the “References” column should read “29-30 & herein”. Please see the corrected Table 1 here.

**Table:** **Table 1.** Influence of *ABCB1* 1199G>A polymorphism on drug transport and/or efficacy.

Compound	1199A activity* (in vitro assay)	Clinical observations in 1199A carriers	References
Rhodamine	= or ↓ (fluorescence)	n.a.	23 & herein
Doxorubicin	= (cytotoxicity)	n.a.	13 & herein
Vinblastine	↑ (cytotoxicity)	n.a.	13 & herein
Vincristine	↑ (cytotoxicity)	n.a.	13
Paclitaxel	↑ (cytotoxicity)	n.a.	13
Etoposide (VP-16)	↑ (cytotoxicity)	n.a.	13
HIV protease inhibitors	↑ (accumulation)	n.a.	24
Cyclosporin A	= or ↑ (accumulation)	Lower concentration in PBMCs	31 & herein
Tacrolimus	↓ (accumulation)	Higher concentration in PBMCs and hepatocytes	29-30 & herein

*Compared to 1199G wild-type; n.a.: not available.

See text for details.

Additionally, there are a few errors with some of the figure legends. Please view Figure 1, Figure 6, and Figure 7 with their corrected legends here.

**Figure 1:**
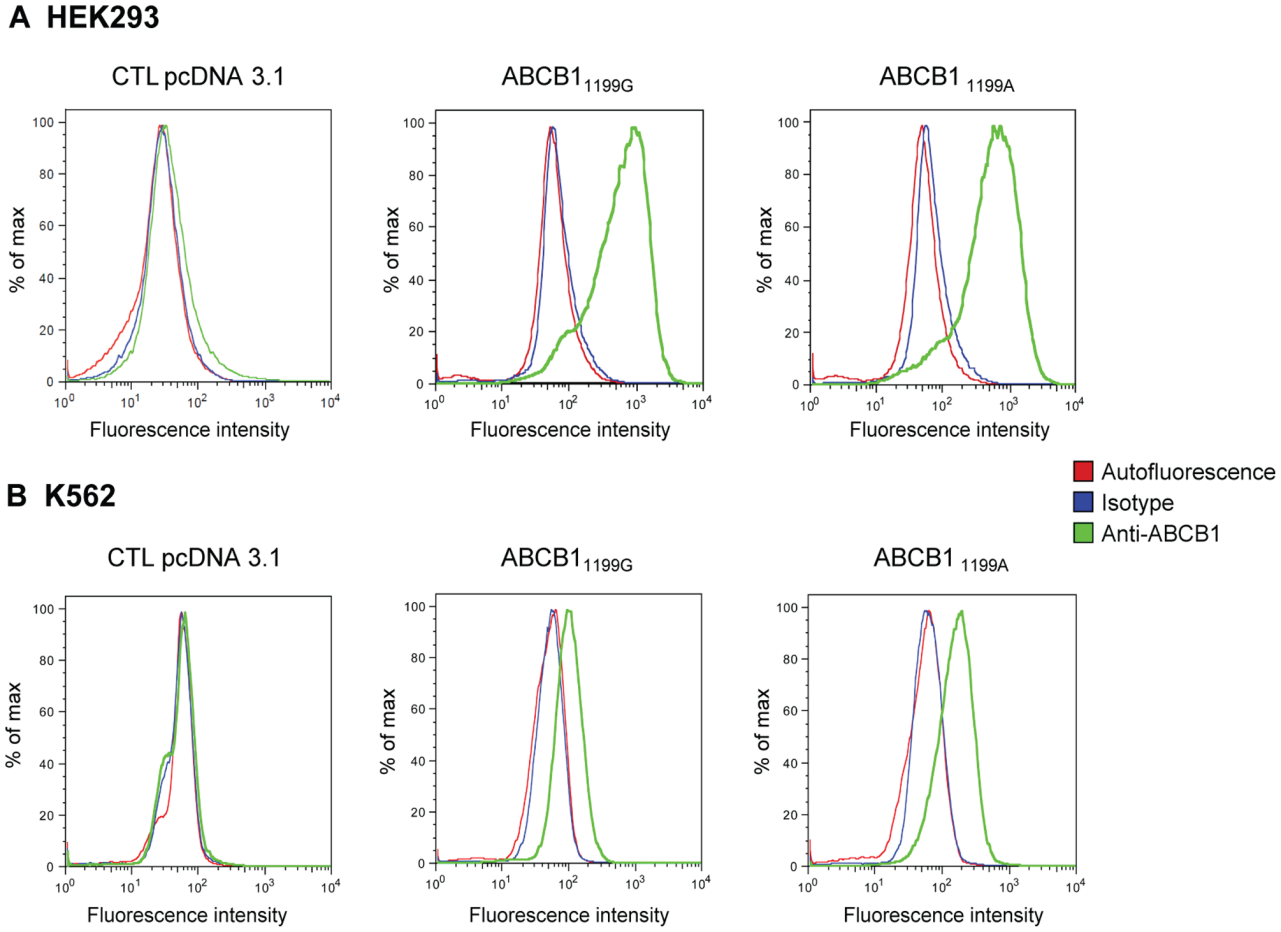
ABCB1 cell surface expression. Histograms generated from a flow cytometry analysis of (**A**) HEK293 transfected with the empty pcDNA3.1 vector (CTL HEK293 pcDNA3.1) and HEK293 cells transfected with *ABCB1*_1199G_ or_ 1199A_ and (**B**) K562 cells transfected with the empty pcDNA3.1 vector (CTL K562 pcDNA3.1), with *ABCB1*_1199G_ or_ 1199A_. Cells were incubated with an FITC-conjugated anti-ABCB1 antibody (green line), a matched isotypic control (blue line) or not labeled (red line).

**Figure 6:**
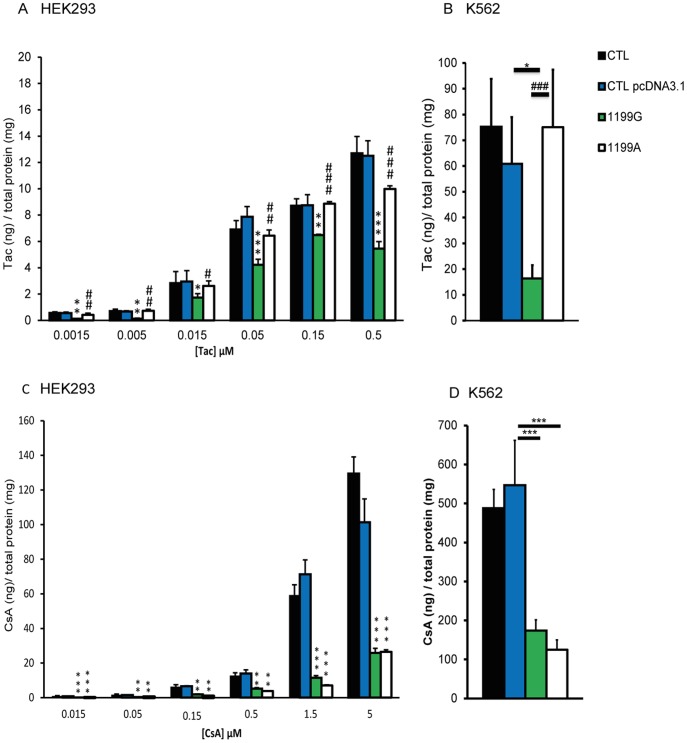
The *ABCB1* 1199A variant differentially affects Tac and CsA efflux. Intracellular accumulation of tacrolimus after 120 min of incubation (N  =  3) (**A**) at six different concentrations (0.0015–0.5 μM) in CTL HEK293, CTL HEKpcDNA3.1, HEK_1199G_ and HEK_1199A_ and (**B**) at 0.05 μM in CTL K562, CTL K562 pcDNA3.1, K562_1199G_ and K562_1199A_. Intracellular accumulation of CsA after 120 min of incubation (N  =  3) (**C**) at six different concentrations (0.015–5 μM) in CTL HEK, CTL HEKpcDNA3.1, HEK_1199G_ and HEK_1199A_ and (**D**) at 0.5 μM in CTL K562, CTL K562pcDNA3.1, K562_1199G_ and K562_1199A_. The intracellular accumulation of Tac or CsA in each cell line was normalized by reporting the absolute amounts of Tac or CsA (in ng) on the total amount of proteins in cell extracts, expressed in mg. * compared to CTL pcDNA3.1 *p<0.05 **p<0.01 ***p<0.001, # compared to 1199G/WT #p<0.05 ##p<0.01 ###p<0.001.

**Figure 7:**
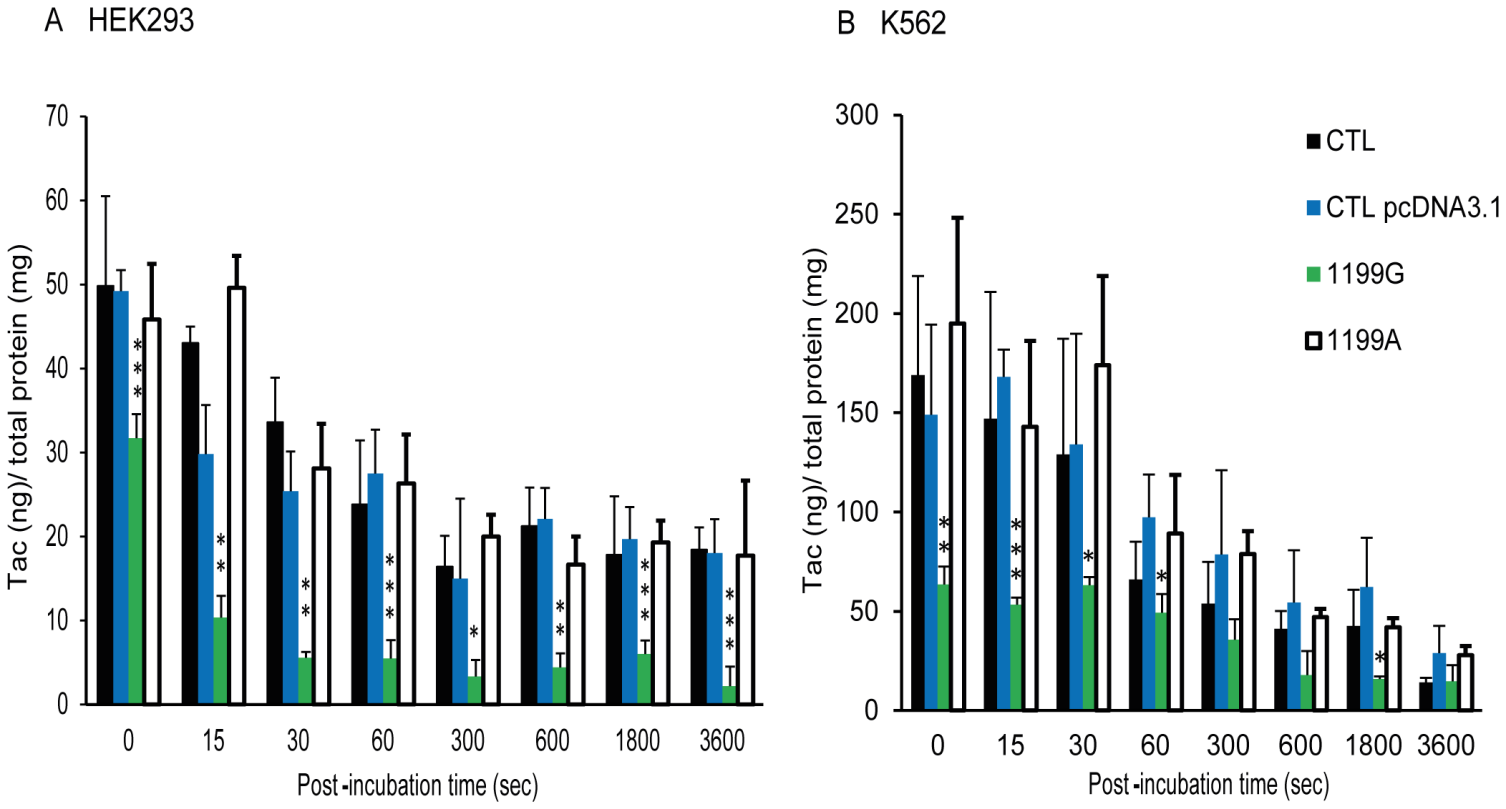
Tacrolimus intracellular kinetics. Intracellular accumulation of tacrolimus (0.05 μM) after 15, 30 sec, 1, 5, 10, 30 or 60 min of efflux (N  =  6) in (**A**) CTL HEK293, CTL HEKpcDNA3.1, HEK_1199G_ and HEK_1199A_ and (**B**) CTL K562, CTL K562pcDNA3.1, K562_1199G_ and K562_1199A_. Cells were pre-loaded with tac for 120 min before efflux. The intracellular accumulation of Tac in each cell line was normalized by reporting the absolute amounts of Tac (in ng) on the total amount of proteins in cell extracts, expressed in mg. * compared to CTL pcDNA3.1 *p<0.05 **p<0.01 ***p<0.001.
